# Longitudinal Mechanism-of-Disease Mapping to Promote Integration Across Disciplines in Preclinical Medical Education

**DOI:** 10.7759/cureus.116540

**Published:** 2026-09-19

**Authors:** Feroza Daroowalla, David M Harris, Nyla Dil, Atsusi Hirumi

**Affiliations:** 1 Medical Education, University of Central Florida College of Medicine, Orlando, USA; 2 Instructional Design, University of Central Florida, Orlando, USA

**Keywords:** concept maps, integration, medical school education, mod maps, pre-clinical medical education

## Abstract

To improve integration of basic science learning and clinical reasoning in preclinical medical education, we developed a longitudinal, case-based activity spanning physiology, immunology, and respiratory pathophysiology. Student teams created mechanism-of-disease maps and illness scripts for respiratory disease cases with increasing complexity across courses. Integration was reinforced through iterative refinement and explicit linkage to previously learned concepts.

The activity was feasible to implement across courses and did not negatively impact student performance on traditional assessments compared to a prior cohort. A standardized rubric enabled consistent evaluation, providing feedback and differentiation of team performance. Student perceptions indicated moderate-to-high engagement, while lower satisfaction scores highlighted areas for improvement related to time constraints and mapping tools.

These findings support the use of longitudinal mapping activities as a scalable approach to promote integration and early clinical reasoning in preclinical medical education.

## Introduction

Clinicians rely on complex, integrated mental models, often described as illness scripts to synthesize patient information and guide diagnostic reasoning [1]. These scripts incorporate risk factors, pathophysiology, clinical presentation, and experiential knowledge. In contrast, novice learners often possess fragmented discipline-specific knowledge, making integration across the basic and clinical sciences challenging. Students require practice in order to integrate basic science concepts to improve their fragmented maps [2].

Although integration is a central goal in medical education, practical, scalable strategies to achieve it remain limited. Basic science disciplines are frequently taught sequentially, leading to compartmentalized learning [3]. Barriers to integration include a lack of available pedagogical tools, time constraints, and inconsistent faculty incentives and buy-in.

Studies highlight that grasping causal mechanisms and connections between basic and clinical sciences can improve diagnostic reasoning [4-7]. Activities that incorporate basic science disciplines into the formation of an illness script can help learners develop a cross-disciplinary conceptual framework similar to that of experts [8,9].

Mechanism-of-disease (MOD) maps provide a structured way to represent causal relationships between underlying mechanisms and clinical manifestations. While previously used in problem-based learning, their longitudinal application across multiple foundational science courses has not been well described [10-12].

The purpose of this study was to evaluate the feasibility of a longitudinal MOD mapping activity across physiology, immunology, and pulmonary pathophysiology. The approach was designed to promote integration through progressive complexity and repeated engagement with shared clinical cases.

## Materials and methods

Study subjects and curricular context

A cohort of 121 medical students (an additional student joined for Course 3) from a class cohort at the University of Central Florida College of Medicine participated in this study over medical school years 1 and 2. The intervention occurred across three courses: physiology, immunology, and pulmonary pathophysiology. Student teams of six students each were assigned by the broader curricular structure for small-group activities, of which these mapping activities were one part. The study was approved by the Institutional Review Board of University of Central Florida. 

Instructional design

The activity was developed by a multidisciplinary team using the 5E instructional model (engage, explore, explain, elaborate, evaluate) [13-15]. This design team included the teaching faculty for the three courses and instructional design consultants. They met regularly to conceive of this sequence of activities and to design the interventions. Case choice and development were done by this team using course objectives, relevance to scaffolding course materials and faculty expertise. The learning management system assignments were designed with the help of educational technologists to allow student teams to upload maps created in freely available map software (Draw.io) and to receive feedback based on the rubric. The teaching faculty came to a shared understanding of how to evaluate students using the rubric they developed.

Design principles included (i) Progression from simple to complex learning; (ii) Use of consistent tools and activities across courses; (iii) Integration of multiple disciplines with scalability to others; (iv) Standardized evaluation using a shared rubric (Table 1); (v) Use of the ARCS IMMS model to evaluate student perceptions of motivation and satisfaction [16-20].

**Table 1:** Evaluation Rubric for MOD Maps The rubric used to evaluate student-generated maps assessed organization, content, and relationships between components.

Category	Exceeds expectations	Meets expectations	Below expectations
Organization	All components (e.g., risk factors, underlying mechanisms, signs and symptoms, treatments) are clearly labeled and easy to distinguish. The map is easy to follow and interpret.	All components are labeled and distinguishable. The map is generally easy to follow.	Components are not clearly labeled or distinguishable. The map is difficult to follow and interpret.
Content	The map is clear and free of misconceptions. It accurately and completely depicts illness script components and all relevant underlying mechanisms.	The map is mostly accurate, with no more than one misconception. It depicts most illness script components and underlying mechanisms.	The map is difficult to follow and includes two or more misconceptions. It fails to depict key illness script components and underlying mechanisms.
Relationships	Relationships between components are clearly depicted and correctly sequenced. Demonstrates strong understanding of underlying disease mechanisms.	Relationships between components are present and mostly correctly sequenced. Demonstrates adequate understanding of underlying mechanisms.	Relationships between components are unclear or incorrectly sequenced. Insufficient detail to demonstrate understanding of underlying mechanisms.

Learning activities

Activities were designed to cohere and build across courses: Course 1 focused on becoming familiar with MOD maps (Figure 1); making maps individually and then in teams on physiology concepts using cases of asthma, infant respiratory distress syndrome (IRDS), chronic obstructive pulmonary disease (COPD) and idiopathic pulmonary fibrosis (IPF). Course 2 expanded on physiology maps to include immunologic mechanisms and relationships. Course 3 introduced illness scripts which incorporate epidemiology, risk factors, diagnostics, pathology, time course and management. Student teams expanded the asthma map and customized the COPD map based on a specific patient encounter video. 

**Figure 1:**
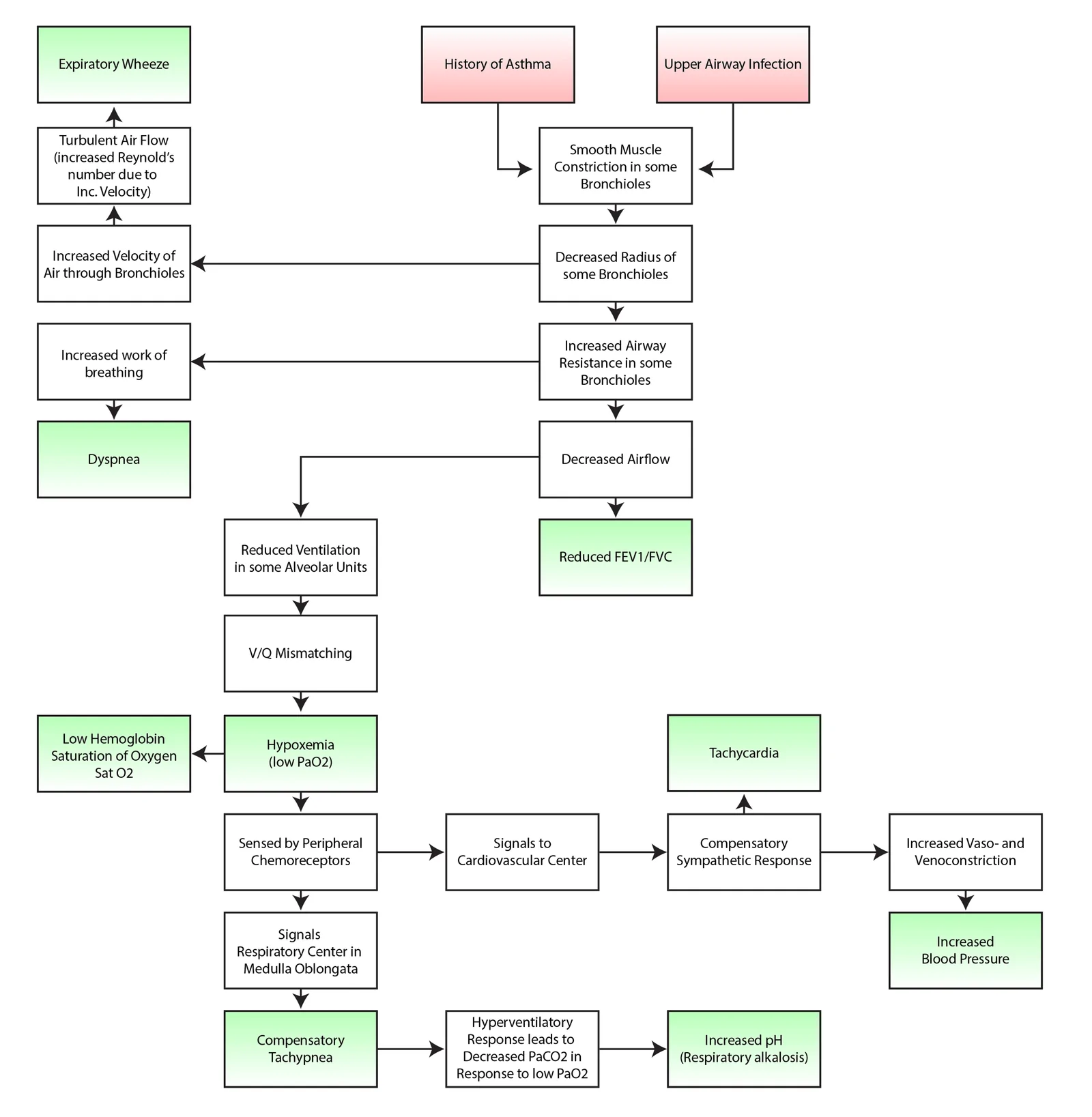
Example of Student-Generated Mechanism-of-Disease (MOD) Map for Physiology Concepts for an Asthma Case

A combination of instructional videos, team activities, and debriefing sessions was mapped to the 5E strategy: (i) Engage: use case vignettes featuring patient signs and symptoms; (ii) Explore: student teams were provided discipline-specific content, instruction, and examples of MOD maps; (iii) Explain: student teams create MOD maps using freely available software; (iv) Elaborate: post-activity debriefing sessions for increasing understanding and incorporating new learning; (v) Evaluation: faculty give students feedback in the learning management system after completion of activity. The rubric was provided to students prior to the learning activities.

Evaluation framework

Outcomes of implementing map-based learning activities across courses were assessed using Kirkpatrick’s model [21]. Level 1 (Student Reaction/Perception) was measured using the Instructional Materials Motivational Survey (IMMS) based on the Attention, Relevance, Confidence, and Satisfaction (ARCS) model. This instrument was distributed at the end of the mapping sessions in each course and included scored questions and areas for written comments. As part of this study protocol and as was feasible during pandemic disruptions, this survey was mandatory in two of the three courses. Level 2 (Student Learning) was evaluated through rubric-based grading of maps and comparison of exam performance with a previous cohort. The rubric assessed organization, content, and demonstration of relationships within the map. Course faculty provided feedback based on whether the map exceeded, met, or fell below expectations.

## Results

Student perceptions (Kirkpatrick Level 1)

Students demonstrated moderate-to-high levels of attention, relevance, and confidence across modules, while satisfaction scores remained consistently lower (Table 2). Relevance scores were the highest across courses, whereas satisfaction was the lowest, suggesting challenges related to workflow and implementation. Internal consistency was high across most domains (Cronbach’s α generally ≥0.7), with the exception of confidence in Course 2. Course 2 had 70% participation in a non-mandatory survey due to COVID-related changes in survey deployment. Qualitative feedback indicated that students valued the integrative and application-based nature of the activity but identified challenges related to time constraints, team dynamics, and mapping software. 

**Table 2:** Student Perception of Mapping Activities Using the ARCS Motivation Model Likert scale: 1: Not true; 2: Slightly true; 3: Moderately true; 4: Mostly true; 5: Very true. α: Cronbach’s alpha.

Domain	Course 1 (N=121) Mean ± SD (α)	Course 2 (N=85) Mean ± SD (α)	Course 3 (N=122) Mean ± SD (α)
Overall ARCS Score	2.98 ± 1.29 (0.94)	3.25 ± 1.25 (0.94)	3.06 ± 1.25 (0.93)
Attention	3.02 ± 1.24 (0.81)	2.95 ± 1.17 (0.81)	2.85 ± 1.23 (0.81)
Relevance	3.42 ± 1.25 (0.78)	3.62 ± 1.22 (0.74)	3.53 ± 1.23 (0.73)
Confidence	3.02 ± 1.29 (0.86)	3.63 ± 1.23 (0.49)	3.38 ± 1.13 (0.70)
Satisfaction	2.46 ± 1.19 (0.88)	2.79 ± 1.14 (0.88)	2.50 ± 1.13 (0.91)

Student learning (Kirkpatrick Level 2)

The rubric effectively differentiated team performance across modules, with improvement observed over time. Fewer teams scored “below expectations” and more teams achieved “meets” or “exceeds” criteria in later modules. For COPD illness scripts in Course 3, no teams received a “below expectations” rating (Table 3). Comparison of exam performance between the study cohort and a previous cohort of students taking the same course and exam items in the prior year showed no statistically significant difference (80.5% vs 77.3%), indicating that the intervention did not negatively impact knowledge acquisition.

**Table 3:** Rubric-Based Performance Across Courses COPD: Chronic Obstructive Pulmonary Disease; IRDS: Infant Respiratory Distress Syndrome; IPF: Idiopathic Pulmonary Fibrosis

Overall grades	Course 1	Course 2	Course 3
Asthma	N=18; Meets: 12, Exceeds: 2, Below: 4	N=20; Meets: 12, Exceeds: 5, Below: 3	N=20; Meets: 13, Exceeds: 4, Below: 3
COPD	N=19; Meets: 8, Exceeds: 3, Below: 8	-	N=20; Meets: 16, Exceeds: 4, Below: 0
IRDS	N=17; Meets: 11, Exceeds: 3, Below: 3	-	-
IPF	N=19; Meets: 10, Exceeds: 4, Below: 5	-	-

## Discussion

This study documents the implementation of iterative mapping activities across Year 1 and Year 2 basic science courses based on respiratory case vignettes that culminated in illness script creation. These activities provided opportunities to integrate physiology, immunology, and pathophysiology within the preclinical curriculum while tracking medical student motivation and perceived relevance throughout the progression. Our outcomes were to deploy this learning activity across courses, give rubric-based feedback including narrative comments about map quality, and understand student perceptions. 

Longitudinal MOD mapping across foundational science courses is feasible and perceived positively by learners. Students showed some evidence of increasingly interconnected knowledge structures without adverse effects on traditional knowledge assessment performance compared with a prior cohort [22]. Mapping and scripting activities enhance integration across basic science disciplines and consequently with clinical knowledge, a process central to the development of clinical reasoning expertise [4,7,11,12]. Serial concept mapping was intentionally designed as a scaffolded learning experience and was predicated on repeating cases of prototypical respiratory disease. The intervention was preceded by content in the discipline, instruction on concept map construction and its educational value, and the evaluation rubric assessing content, organization, and relationships. The goal was to expand existing cognitive frameworks by helping learners integrate prior knowledge and explicitly articulate the underlying basic science concepts while analyzing clinical information [23,24]. Scores on team maps in our cohort point to progressive integration of underlying mechanisms and inter-relationships [22,23,25], even though we did not measure application in other settings. 

The intervention was achievable within our existing curriculum, which combines lectures and case-based team learning, and provided a valuable complement to other instructional approaches [22,23,26]. It was the only activity that spanned multiple courses, with teams refining and expanding prior maps while incorporating insights from faculty-generated maps provided at the conclusion of each course. Team-based mapping promotes collaborative learning and shared meaning-making [27] while facilitating faculty feedback. Rubric-based qualitative and narrative feedback enabled meaningful faculty interaction with student teams. The rubric effectively differentiated performance across content, organization, and conceptual relationship domains and rewarded highly interconnected maps, which are associated with deeper learning than hierarchical or spoke-and-wheel structures [28]. Rubric assessment was formative in all three courses.

Evaluation using the ARCS framework provided insight into student engagement and motivation. Students reported moderate to high levels of attention, relevance, and confidence across courses. Relevance ratings were particularly high when activities incorporated patient cases and clinical videos, underscoring the value of authentic clinical context. Confidence increased over time, suggesting that repeated exposure supported students’ ability to integrate and apply knowledge while increasing familiarity with the mapping process. Satisfaction scores were lower, reflecting challenges related to time demands, mapping software, and team coordination. Learners are more receptive to concept mapping when given adequate orientation to both the method and its rationale, which we incorporated [23,29]. Student feedback pointed to additional implementation improvements, including software usability, setting clear expectations, and giving sufficient time for collaboration. 

Several limitations should be considered. The study was conducted at a single institution and involved one class cohort, limiting generalizability. The absence of a contemporaneous control group restricts causal inference. Outcomes were assessed primarily at the team level and did not evaluate individual student performance. In addition, rubric scoring was conducted by a single rater, limiting assessment of interrater reliability and introducing the potential for bias. Finally, higher-level outcomes like transfer to clinical performance and diagnostic reasoning were not assessed. 

## Conclusions

This study contributes to the literature by demonstrating the feasibility of applying MOD mapping longitudinally across multiple courses with increasing complexity. This approach may help learners transition from fragmented knowledge to more integrated, expert-like mental models and may support early development of clinical reasoning skills. Longitudinal use of MOD maps and illness scripts is a feasible and scalable strategy for integrating basic science concepts in preclinical medical education. This approach promotes engagement, supports early development of clinical reasoning frameworks, and maintains learning performance.

Future work should evaluate implementation across additional disciplines and examine long-term effects on clinical reasoning and diagnostic accuracy. Incorporation of individual-level assessment and longitudinal tracking may provide further insight into the development of integrated knowledge structures. Further research should explore long-term educational impact and application in clinical training.
